# Established and emerging GABA_A_ receptor pharmacotherapy for epilepsy

**DOI:** 10.3389/fphar.2024.1341472

**Published:** 2024-02-21

**Authors:** Robert J. Richardson, Steven Petrou, Alexander Bryson

**Affiliations:** ^1^ Florey Institute of Neuroscience and Mental Health, University of Melbourne, Melbourne, VIC, Australia; ^2^ Department of Neurology, Austin Health, Heidelberg, VIC, Australia; ^3^ Praxis Precision Medicines, Boston, MA, United States; ^4^ Department of Neurology, Eastern Health, Melbourne, VIC, Australia

**Keywords:** benzodiazepines, epilepsy, GABA A receptor, GABA allosteric modulators a, neurosteriod, antisense, oligonucleotide-drug conjugates

## Abstract

Drugs that modulate the GABA_A_ receptor are widely used in clinical practice for both the long-term management of epilepsy and emergency seizure control. In addition to older medications that have well-defined roles for the treatment of epilepsy, recent discoveries into the structure and function of the GABA_A_ receptor have led to the development of newer compounds designed to maximise therapeutic benefit whilst minimising adverse effects, and whose position within the epilepsy pharmacologic armamentarium is still emerging. Drugs that modulate the GABA_A_ receptor will remain a cornerstone of epilepsy management for the foreseeable future and, in this article, we provide an overview of the mechanisms and clinical efficacy of both established and emerging pharmacotherapies.

## 1 Introduction

Epilepsy is a common neurological disorder characterised by an excess of excitatory activity within neural circuits and a predisposition to seizures. GABA is the primary inhibitory neurotransmitter in the mammalian brain and therefore plays a central role in the maintenance of normal excitation-inhibition balance and the pathophysiology of epilepsy.

GABA exerts much of its inhibitory influence through GABA_A_ receptors which comprise a diverse group of ligand-gated ion channels that serve as common targets for antiseizure medications (Perucca et al., 2023a; Perucca et al., 2023b; Bryson et al., 2023). Indeed, some of the earliest developed antiseizure medications such as potassium bromide and phenobarbital act primarily through positive allosteric modulation of the GABA_A_ receptor and, more recently, there has been great interest in the development of novel compounds that precisely modulate inhibitory pathways through specific GABA_A_ receptor subtypes (Engin et al., 2018; Owen et al., 2019; Jankovic et al., 2021; Cerne et al., 2022; Witkin et al., 2022). The development of these novel compounds has emerged from a growth in understanding of the structure and function of GABA_A_ receptors and insights into complex inhibitory effects mediated by receptor subtypes exhibiting variation in cellular location and regional distribution.

In this mini review we will first provide a brief overview of key aspects of the GABAergic system most relevant to pharmacological modulation in epilepsy, and then discuss both long-standing and newer or emerging drugs whose primary mechanism of action is via modulation of GABA_A_ receptor neurotransmission.

## 2 Background: GABA_A_ receptor transmission and relevance in epilepsy

### 2.1 Structure and distribution of GABA_A_ receptors

GABA_A_, GABA_B_ and GABA_C_ receptors have been well characterised and form the basis of GABAergic neurotransmission in the mammalian nervous system. Both GABA_A_ and GABA_C_ receptors are ligand gated ion channels, with the latter being largely confined to the retina and possessing no known role in the pathogenesis or pharmacological management of epilepsy (Enz and Cutting, 1999; Sieghart et al., 1999; Olsen and Sieghart, 2009; Sigel and Steinmann, 2012). GABA_B_ receptors are G-protein coupled receptors that exert both post and pre-synaptic effects, in part through activation of inward-rectifying K^+^ channels and hyperpolarisation of the neuronal membrane (Bowery et al., 2002). No established antiseizure medications, and comparatively few drugs in wider clinical use, act primarily through modulation of the GABA_B_ receptor. GABA_A_ receptors, the focus of this review, are cys-loop heteropentameric ligand gated ion channels permeable to chloride and bicarbonate (Chuang and Reddy, 2018; Bryson et al., 2023). The subunits comprising the quaternary structure are denoted α-, β-, δ-, γ-, π-, θ-, ρ-, ε-, with genes encoding these subunits located on chromosomes 4, 5, 15, and X (Chuang and Reddy, 2018). To date, 19 subunits have been cloned termed α1-6, β1-3, δ1, γ1-3, π1, θ, ρ1-3 and ε1, which are assembled as heteropentamers within the endoplasmic reticulum before being trafficked to the neuronal cell membrane (Sieghart et al., 1999; Sigel and Steinmann, 2012; Chuang and Reddy, 2018). Given the number of possible subunit arrangements, there is potential for great diversity of GABA_A_ receptors possessing a range of physiological and pharmacological properties. The majority of GABA_A_ receptors in the mammalian brain are composed of α1β2γ2 subunits (50%–60%) with the next most prevalent (25%–35%) being composed of combinations of α1 or 2, β1,2 or 3 and γ2 (Olsen and Sieghart, 2008; 2009).

α1β2γ2 GABA_A_ receptors are located within the synaptic cleft and possess rapid activation and desensitisation kinetics upon GABA binding which are ideal for fast neurotransmission (Gingrich et al., 1995). Although α2 and α3 containing receptors are also concentrated within the synaptic cleft they have differing regional and cellular distributions which carries relevance for subunit-specific drug development. GABA_A_ receptors distributed within the extra-synaptic membrane tend to contain α4, α5 and α6 subunits and possess slower activation kinetics and incomplete desensitisation upon GABA binding (Glykys and Mody, 2007). α4 and α6 subunits preferentially co-assemble with δ-subunits, and these receptors are emerging as an important pharmacological target given their sensitivity to neurosteroids and their diverging physiological role compared to synaptic receptors (MacKenzie and Maguire, 2013).

Recently, a number of protein complexes that co-localise and interact with ligand-gated ion channels, including the GABA_A_ receptor, have been identified (Maher et al., 2017). The auxillary subunits LHFPL4 (Davenport et al., 2017) and Cltpm1 (Ge et al., 2018) have been shown to regulate the stabilisation and trafficking of post-synaptic GABA_A_ receptors, respectively, and Shisa7 interacts with α1, α2 and γ2 containing GABA_A_ receptors to ehance trafficking and expression at synaptic and extrasynaptic sites (Han et al., 2019; Han et al., 2021). Shisa7 has also been shown to modulate GABA_A_ receptor decay kinetics and, interestingly, enhance diazepam-potentiated inhibitory currents in hippocampal neurons. Although it is currently unknown if Shisa7 exerts a similar influence upon other compounds acting upon the GABA_A_ receptor, is raises the intriguing possibility that auxiliary subunits may serve as future pharmacological targets.

### 2.2 Phasic and tonic GABAergic transmission

GABA_A_ receptors mediate two main forms of neurotransmission which are subject to pharmacological modulation: phasic and tonic transmission. Phasic transmission occurs when GABA released from presynaptic axon terminals bind GABA_A_ receptors clustered in the post-synaptic membrane. This results in a spatially precise and rapid change in chloride conductance which, due to higher postnatal expression of the potassium-chloride cotransporter, usually causes hyperpolarisation of the post-synaptic membrane in mature neurons (Farrant and Nusser, 2005; Farrant and Kaila, 2007; Kaila et al., 2014). At the cellular level phasic inhibition can exert multiple effects on neuronal function determined by the subcellular location of GABA_A_ receptors and variation of the intracellular chloride gradient (Farrant and Nusser, 2005). For example, dendritic inhibitory inputs can suppress dendritic spikes and backpropagating action potentials and regulate coincidence detection of excitatory inputs (Tang et al., 2011; Groen et al., 2014). In contrast somatic and axonal inputs may co-ordinate spike timing and pyramidal cell synchronisation (Tremblay et al., 2016). The presence of GABA_A_ receptor subunit variation across subcellular compartments raises the possibility of targeted pharmacological modulation of these physiological processes.

Tonic inhibition is a more persistent form of neurotransmission mediated by extracellular GABA activating extra-synaptic GABA_A_ receptors. As such, tonic inhibition exerts a more non-specific inhibitory influence upon both neurons and glia and across subcellular regions including the axon, dendrites and cell body (Farrant and Nusser, 2005). Tonic inhibition plays a crucial role in normal brain development (Valeyev et al., 1993; LoTurco et al., 1995; Owens et al., 1999; Demarque et al., 2002) and, in the mature CNS, has been shown to exert an important modulatory role within the hippocampus (Semyanov et al., 2003), cerebral cortex (Vardya et al., 2008), thalamocortical relay networks (Porcello et al., 2003), midbrain (Tossell et al., 2021) and cerebellum (Hausser and Clark, 1997). Despite the diffuse actions of tonic inhibition more targeted effects may arise through localised extracellular GABA release from neurogliaform cells and cellular variation in extrasynaptic GABAA receptor subtype expression which may also be leveraged through pharmacologic manipulation (Olah et al., 2009; Tremblay et al., 2016).

### 2.3 GABA_A_ receptor mutations and epilepsy

The relevance of GABA_A_ neurotransmission in both the pathophysiology and treatment of epilepsy is underscored by the discovery of an expanding number of GABA_A_ receptor mutations associated with forms of genetic epilepsy. These discoveries have also raised interesting pharmacotherapeutic issues. Mutations of the γ2 subunit (encoded by the *GABRG2* gene) were the earliest identified and have since been comprehensively examined (Baulac et al., 2001; Wallace et al., 2001). These loss of function monogenic variants have been shown to cause developmental and epileptic encephalopathies (DEEs) including Dravet Syndrome and Lennox-Gastaut Syndrome, in addition to milder phenotypes such as Febrile Seizures and Genetic Generalized Epilepsies (Baulac et al., 2001; Wallace et al., 2001; Harkin et al., 2002; Kananura et al., 2002; Tian et al., 2013; Huang et al., 2014; Todd et al., 2014; Kang et al., 2015; Warner et al., 2019; Qu et al., 2021). Since the γ2 subunit contributes to the benzodiazepine binding site these variants may be associated with benzodiazepine insensitivity which carries implications for appropriate drug treatment (Fedi et al., 2006). Mutations of *GABRA1/2/3/5*, *GABRB1/2/3* and *GABRD,* which encode the α1,2,3,5, β1,2,3 and δ subunits, respectively, have also been implicated in a spectrum of epilepsies, including severe DEEs, although variants of *GABRA1* and *GABRB2/3* mutations are most frequently encountered which likely reflects the prevalence of their expression in the CNS (Absalom et al., 2023). Interestingly, although most GABA_A_ receptor variants show loss-of-function traits, characterization of several β3, δ and α4 variants revealed gain-of-function features including enhanced sensitivity to GABA and were associated with more severe early-onset phenotypes (Komulainen-Ebrahim et al., 2019; Mierzewska et al., 2021; Absalom et al., 2022; Ahring et al., 2022; Johannesen et al., 2022; Maillard et al., 2022) Importantly, the discovery of gain-of-function mutations carries treatment implications as drugs which enhanced GABA_A_ receptor transmission tended to be more effective in loss-of-function variants.

## 3 Established GABA_A_ receptor pharmacotherapy in epilepsy.

Drugs that act on GABA_A_ receptors have been used for the management of epilepsy for over a century and remain important treatment tools for acute and chronic seizure control (Rho and White, 2018).

### 3.1 Phenobarbital

Phenobarbital is a barbiturate and sedative-hypnotic agent, and one of the earliest discovered antiseizure medications introduced in 1912. Phenobarbital enhances both phasic and tonic inhibition through positive allosteric modulation of GABA_A_ receptors and exhibits minimal subunit specificity. It has a long half-life of 75–120 h, is ∼25% renally excreted and is a broad hepatic enzyme inducer which is of relevance for administration in the intensive care setting in the context of status epilepticus and polypharmacy (Brodie and Kwan, 2012). Phenobarbital is used in both focal and generalised epilepsies and, in two meta-analyses, was found to have similar efficacy to both phenytoin and carbamazepine for time to 12-month seizure remission and first breakthrough seizure in these conditions, although carbamazepine was associated with lower rates of seizure recurrence and drug cessation (Nolan et al., 2013). Chronic use of phenobarbital is limited by concerns regarding sedation and cognitive adverse effects; however it retains an important role in resource-limited settings (Brodie and Kwan, 2012). Phenobarbital also has an established role in refractory status epilepticus (SE). In benzodiazepine-resistant SE, a systematic review and network meta-analyses suggested that phenobarbital was more effective for terminating seizures compared to phenytoin, lacosamide, valproate and levetiracetam (Brigo et al., 2022), and as first-line therapy it was found to have comparable efficacy to lorazepam (Treiman et al., 1998). Again, however, the use of phenobarbital in acute management is limited by adverse effects, including hypotension and respiratory depression, and a slow rate of administration, and it is often reserved as third-line therapy for SE in the intensive care setting.

### 3.2 Benzodiazepines

Benzodiazepines (BZDs) are widely prescribed for the acute management of seizures and status epilepticus. They are used less frequently as add-on therapy for the management of refractory chronic epilepsy. BZDs act as positive allosteric modulators of the GABA_A_ receptor and bind at the interface of the γ2 and α1, α2, α3 or α5 subunits. Given the preferential localisation of γ2 subunit containing receptors at the post-synaptic membrane they augment phasic inhibition but have minimal effect on tonic inhibition (Benson et al., 1998).

The 1,4-benzodiazepines lorazepam, midazolam, and diazepam are used for a variety of clinical indications, including procedural sedation, anxiety, and alcohol withdrawal, and have established efficacy for the suppression of seizures (Kienitz et al., 2022). In addition to oral and intravenous formulations, midazolam and lorazepam are available as intranasal formulations, and midazolam as intramuscular and buccal formulations. Both lorazepam and diazepam have high oral bioavailability (over 90%) compared to midazolam (∼40–50%, due to metabolism in the intestinal epithelium) with peak concentrations reached between 30 and 120 min (Kienitz et al., 2022). Faster peak concentrations are achieved with buccal (∼30 min) and intranasal (7–15 min) midazolam, and so these routes have been investigated for the out-of-hospital management of seizures. Importantly, benzodiazepines have greatly varying half-lives with midazolam shortest (1.5–3 h) followed by lorazepam (8–25 h) and diazepam (24–48 h) (Kienitz et al., 2022). Comparative studies in the management of SE suggest that intravenous lorazepam is more effective than intravenous diazepam for pre-hospital termination of seizures (Alldredge et al., 2001) and a comparison of intravenous lorazepam to intramuscular midazolam found higher rates of seizure cessation on arrival to hospital with midazolam (Silbergleit et al., 2012) likely due to faster rates of administration. Finally, buccal midazolam has been associated with faster rates of out-of-hospital seizure termination compared to rectal diazepam in paediatric and residential care patients (Scott et al., 1999; Nakken and Lossius, 2011) and intranasal midazolam has been found to have comparable effectiveness to intravenous diazepam for terminating seizures in the paediatric population (Lahat et al., 2000; Mahmoudian and Zadeh, 2004).

Clobazam is a 1,5-benzodiazepine and possesses a different molecular structure to classical 1,4- benzodiazepines such as diazepam. This confers greater selectivity to GABA_A_ receptors possessing the α2 subunit which may be implicated in the anticonvulsant effects of BZDs, and is associated with other favourable properties such as improved tolerability (Stephens et al., 2017; Engin, 2022). Consequently, clobazam has a more established role in the long-term management of epilepsy, with robust evidence in the treatment of the DEE Lennox-Gastaut Syndrome. In a randomised phase 2 trial clobazam exhibited a dose-dependent reduction in the frequency of atonic seizures with reductions of non-drop seizures also observed in the high-dose (1 mg/kg/day) group Kienitz et al., 2022 #292}and, in an open label extension, it was found that in over 80% of patients who had a favourable response this benefit was sustained by year three (Conry et al., 2014). Although trial evidence is limited, clobazam also appears to have efficacy as add-on therapy in the management of refractory focal epilepsy. The improved tolerability of clobazam has motivated the search for more targeted positive allosteric GABA_A_ modulators to mitigate the side effects associated with classical benzodiazepine.

### 3.3 Vigabatrin

Vigabatrin is a structural analogue of GABA and was developed in 1974 using a rational drug design approach to inhibit GABA breakdown through the targeting of GABA-transaminase. This raises the intracellular concentration of GABA and leads indirectly to enhanced phasic and tonic GABA_A_ receptor transmission by augmenting both vesicular release and extra-synaptic extrusion via GABA-transporters (GATs) (Ben-Menachem, 2011). Vigabatrin has a short half-life (5–7 h) and achieves low CSF concentrations, but, since it acts as an irreversible GABA-transaminase inhibitor, it is biological effect of increasing GABA levels within the brain can persist for over 1 week. Vigabatrin has efficacy in both infantile spasms and as add-on therapy for focal seizures in adults. It has been shown to rapidly decrease the frequency of infantile spasms within 5 days compared to placebo (Appleton and Montiel-Viesca, 1993; Appleton et al., 1999) and exhibits a dose-dependent increase in spasm cessation (Elterman et al., 2001; Elterman et al., 2010). Similar findings have been observed in adults with refractory focal seizures, with several randomised controlled trials showing a dose-dependent decrease in the number of median monthly seizures, and an increase in the proportion of subjects with over 50% seizure reduction (French et al., 1996; Dean et al., 1999). A particular concern with vigabatrin use is the development of an irreversible peripheral visual field defects. This occurs with a prevalence of 20%–25% in adults and approximately 15% in children, with onset observed within 9 months after commencement.

### 3.4 Tiagabine

Like vigabatrin, tiagabine indirectly enhances GABA_A_ receptor transmission, and was strategically developed in the 1990s to act as a GAT blocker thereby enhancing the availability of GABA at the synaptic cleft. Tiagabine exhibits approximately 2.5-fold greater specificity for GAT1 which mediates glial and neuronal reuptake in the vicinity of the synaptic cleft which reflects its physiological impact of prolonging the duration of inhibitory post-synaptic currents. Tiagabine is metabolized through the CYP3A4 system and has a half-life of 5–9 h which is shortened with the concomitant use of hepatic enzyme inducing agents. Several studies have confirmed the benefit of tiagabine in refractory focal epilepsy in adults, including a placebo-controlled cross-over trial demonstrating a 54% response rate (over 50% reduction in seizure frequency) compared to 24% for placebo (Richens et al., 1995), and moderate-to-high doses of tiagabine (32 and 56 mg/day) showing greater response than low dose (16 mg/day) and placebo (Uthman et al., 1998). In the paediatric population there is also evidence for efficacy in focal epilepsy but there is the possibility of exacerbating certain generalised seizures types, including myoclonic seizures and primary generalised tonic-clonic convulsions (Uldall et al., 2000). Adverse effects, including fatigue and dizziness, and concerns raised about provoking seizures, in particular absence seizures associated with generalised spike-wave discharges, have limited its clinical use.

### 3.5 Valproate

Although valproate possesses several mechanisms of action, including sodium channel blockade, modulation of excitatory neurotransmitters and inhibition of histone deacetylase, it is thought to achieve its anti-seizure effects at least in part through augmenting GABA_A_ transmission (Davies, 1995), and so will be considered in further detail. Valproate does not appear to act directly on GABA_A_ receptors but has shown to increase brain GABA levels through several pathways, including preferential inhibition of GABA-transaminase within neurons and enhancing glutamate decarboxylase (GAD) activity (Luder et al., 1990; Loscher, 1999). It is possible that valproate may also enhance GABA synaptic release and modulate metabolic pathways, such as the inhibition of alpha-ketoglutarate, to increase activity through the GABA shunt. Valproate has a half-life of 9–18 h and is metabolized through the hepatic CYP450 system and glucuronidation, and co-administration with hepatic enzyme inducers shorten its half-life. It is highly protein bound and inhibits hepatic metabolism, both of which can increase the levels of other antiseizure medications including lamotrigine, phenytoin, carbamazepine, and phenobarbital. Valproate has efficacy in both focal and generalised forms of epilepsy, and in the Standard and New Antiepileptic Drugs (SANAD) trial, which compared valproate to lamotrigine and topiramate in generalized and unclassified epilepsy, it was found to be more effective than lamotrigine for 12-month seizure remission, and superior to topiramate for time-to-treatment failure (Marson et al., 2007). The SANAD 2 trial compared valproate to levetiracetam as first-line treatment in patients with genetic/idiopathic generalised epilepsy, demonstrating superiority of valproate for both 12-month remission and time to first seizure (Marson et al., 2021). In patients with childhood absence epilepsy, valproate has been shown to have superior efficacy to lamotrigine, and is equivalent to ethosuximide, although was associated with higher rates of attentional deficits (Glauser et al., 2010). Despite its efficacy in a broad range of syndromes, a significant concern relates to teratogencitiy with high rates of congenital malformation and autism, and lower IQ in exposed infants necessitating careful counselling.

## 4 Emerging and novel GABA_A_ receptor pharmacotherapy

### 4.1 Cenobomate

Cenobomate is a newer antiseizure medication that recently gained FDA and European Medicines Agency approvals for the treatment of refractory focal onset seizures in adults (Roberti et al., 2021). Cenobomate modulates both GABA_A_ receptors (Sharma et al., 2020) and voltage gated sodium channels, with a preferential effect on channel inactivation leading to a reduction of the persistent sodium current (Nakamura et al., 2019). Cenobomate acts as a positive allosteric modulator of GABA_A_ receptors through non-benzodiazepine binding sites. Although cenobomate acts upon both synaptic and extra-synaptic GABA_A_ receptors, the half maximal effective concentration in a non-neuronal expression system was higher for α4, α5 and α6 containing GABA_A_ receptor subtypes which are associated with extrasynaptic localisation, and in dissociated CA1 neurons cenobomate exerted a more pronounced effect on baseline GABA holding current than spontaneous inhibitory post synaptic currents. Together, these findings suggest that cenobomate preferentially modulates of tonic inhibition (Sharma et al., 2020).

In two randomised placebo-controlled Phase 2 trials in patients with refractory focal seizures, cenobomate at doses of 200 or 400 mg per day were associated with over 50% reduction in seizure frequency during treatment maintenance compared to placebo (Chung et al., 2020; Krauss et al., 2020; Rosenfeld et al., 2021). Notably, high rates (11%–26%) of seizure-freedom were also observed at these doses, and a *post hoc* analyses of two open-label phase 3 trials suggest that responder and seizure-free rates persist beyond 12 months (Aboumatar et al., 2022; Rosenfeld et al., 2021). A more recent cohort study in highly active (over 20 seizures/month) or ultra-refractory patients (over six anti-seizure medication failures), most of whom had undergone previous epilepsy surgery or vagal-stimulators insertion, showed clinically significant reductions seizure severity and frequency (Pena-Ceballos et al., 2023). Five percent of this cohort achieved seizure freedom and 70% of patients had a 50%–99% reduction in seizure frequency, most of whom were treated with over 250 mg of cenobomate (Pena-Ceballos et al., 2023).

Dose related adverse effects from cenobomate include somnolence, dizziness, fatigue, and coordination difficulties. Interestingly, adverse effects were more frequently reported in a patient cohort that was concurrently treated with a sodium channel blocking drug, perhaps due to the overlapping mechanism of action (Krauss et al., 2020). A rare severe side effect of Drug Rash with Eosinophilia and Systemic Symptoms (DRESS) was observed in one of the phase 2 trials (Krauss et al., 2020) but in studies using a slower dose titration no further cases were identified (Krauss et al., 2020; Lattanzi et al., 2020).

### 4.2 Darigabat

Darigabat is an imidazopyridine-related molecule that was developed to act selectively upon GABA_A_ receptors containing α2,α3 and α5 subunits (Owen et al., 2019). Darigabat is a positive allosteric modulator and binds the benzodiazepine site of α1, α2, α3, and α5 subunit containing GABA_A_ receptors, but its efficacy at α1-containing receptors is weak which may reduce unwanted side effects such as sedation and abuse potential whilst maintaining antiseizure and anti-anxiolytic properties which are be mediated primarily through α2 and α3-containing receptors (Engin et al., 2018; Nickolls et al., 2018; Owen et al., 2019; Cerne et al., 2022).

Darigabat has shown antiseizure efficacy in a range of preclinical epilepsy models in animals, including the kainic acid model of mesial temporal lobe epilepsy where it produced a statistically significant reduction in hippocampal paroxysmal discharges with comparable efficacy to diazepam (Owen et al., 2019; Bialer et al., 2020; Gurrell et al., 2022). Phase 1 clinical trials demonstrated safety and tolerance with only mild somnolence and dizziness reported in subjects (Nickolls et al., 2018; Bialer et al., 2022). In a small study of patients with photosensitive epilepsy, darigabat abolished the photoparoxysmal response in 6 of 7 patients following single doses between 17.5mg and 52 mg (Gurrell et al., 2019). There was no difference in suppression of photoparoxysmal responses between lower and higher doses of darigabat and efficacy was similar to that of lorazepam (Gurrell et al., 2019). Currently a large multicentred Phase 2 trial is underway (NCT04244175) to assess the safety and efficacy of darigabat as adjunctive therapy for refractory focal and generalised seizures, the results of which are unpublished (Cerne et al., 2022; Gurrell et al., 2022).

### 4.3 Padsenovil

Padsenovil was developed out of a rational drug discovery program to modulate both synaptic vesicle protein 2 (SV2) and GABA_A_ receptors, thereby exerting an effect on both pre-synaptic and post-synaptic targets (Niespodziany et al., 2020). At the GABA_A_ receptor, padsevonil binds at the benzodiazepine site and acts as a positive allosteric modulator and partial agonist which may reduce complications associated with full agonists (Leclercq et al., 2020). *In vitro* studies demonstrated greatest potency on α1 and α5 compared to α2 and α3 subunit containing receptors (Wood et al., 2020). Padsevonil showed promising results in several acute and chronic seizure models, including greater protection from seizures in the chronic 6 Hz seizure model compared to diazepam and the existing SV2 modulators levetiracetam and brivaracetam, and in a Phase 2 proof-of-concept trial showed significant reductions in weekly seizure frequency for treatment-resistant focal epilepsy. However, a randomised dose-finding trial and Phase 3 trial failed to observe a significant reduction in seizure frequency at any dose compared to placebo leading to discontinuation of drug development (Rademacher et al., 2022).

### 4.4 Alprazolam

Alprazolam is a short-acting and non-selective 1,4 benzodiazepine with an established role in the treatment of anxiety disorders. Despite animal studies showing potent antiseizure effects comparable to other benzodiazepines, alprazolam has historically not been used for the management of acute seizures (Jenck et al., 1992; De Sarro et al., 1996) and is unsuitable for the treatment of chronic epilepsy due to tolerance and drug dependence. However, alprazolam is being repurposed as an acute rescue medication for seizures in the outpatient setting via inhalation with the Staccato device as it achieves rapid onset of action combined with a relatively convenient administration route (French et al., 2019). In a proof-of-concept study, inhaled alprazolam supressed the photoparaxysmal response on electroencephalography within minutes and maintained its effects for up to 4 hours (French et al., 2019), and in a randomised trial of hospital inpatients Staccato alprazolam led to higher rates of seizure termination within 2 minutes compared to placebo (French et al., 2023). A phase 3 trial to test efficacy in the outpatient setting is now recruiting.

### 4.5 Stiripentol

Stiripentol is a structurally unique antiseizure medication that has been shown to modulate GABA_A_ receptors in addition to several other secondary mechanisms, including inhibition of lactate dehydrogenase (LDH) (Nickels and Wirrell, 2017). Stiripentol acts independently of the benzodiazepine binding site, promotes increased GABA_A_ receptor open time duration and its effects are blocked by phenobarbital, which together suggest a barbiturate-like effect (Quilichini et al., 2006). Stiripentol acts upon both synaptic and extrasynaptic GABA_A_ receptor subtypes and has a propensity for α3 subunit-containing receptors which are expressed during embryonic and early post-natal development (Nickels and Wirrell, 2017). It enhances the delay constant of inhibitory post-synaptic potentials and augments tonic inhibition via activation of δ-containing receptors (Nickels and Wirrell, 2017). Stiripentol undergoes extensive hepatic metabolism and drug levels are reduced with co-administration of enzyme-inducing antiseizure medications such as phenytoin, carbamazepine and phenobarbital.

Stiripentol has efficacy in paediatric forms of epilepsy, in particular the severe developmental and epileptic encephalopathy Dravet Syndrome. Following promising results in an open-label adjunctive-therapy study, two placebo-controlled studies demonstrated impressive responder rates (over 50% reduction in seizure frequency) of 71% and 67% compared to placebo, and although the study sizes were small a significant proportion of participants achieved seizure freedom. In an open-label extension responder rates were maintained at over 50% (Chiron et al., 2000) although an important consideration is that clobazam, which also has benefit in Dravet syndrome, can increase stiripentol levels by inhibiting hepatic metabolism. Stirpentol also appears to carry benefit as adjunct therapy in childhood focal epilepsies and several case studies suggest a potential role as rescue therapy in refractory status epilepticus which may relate to modulation of extra-synaptic GABA_A_ receptors (Nickels and Wirrell, 2017).

### 4.6 Neurosteroids

Neurosteroids such as allopregnanolone, allotetrahydrodeoxycorticosterone (THDOC) and androstanediol are endogenously produced metabolites of the steroid hormones progesterone and corticosterone (MacKenzie and Maguire, 2013). Neurosteroids are synthesised within the brain and fluctuations in their concentration are linked to physiological states such as the menstrual cycle, the *postpartum* period and increased stress states (Purdy et al., 1991; MacKenzie and Maguire, 2013; Reddy and Estes, 2016; Chen et al., 2019). There is also evidence that they play a pivotal role in several neuropsychiatric disorders including anxiety, pre-menstrual dysphoric disorder, anxiety (MacKenzie and Maguire, 2013; Sikes-Keilp and Rubinow, 2023) and epilepsy (Reddy, 2013).

Neurosteroids are potent GABA_A_ receptor positive allosteric modulators and bind within the transmembrane domain of α and β subunits to activate both synaptic and extrasynaptic receptors (Hosie et al., 2007; Chen et al., 2019). δ-subunit containing receptors that mediate tonic inhibition are particularly sensitive to neurosteroids, and this mechanism of action has attracted significant interest for a potential role in refractory epilepsy and status epilepticus as there is evidence for synaptic receptor downregulation in these conditions (Belelli et al., 2002; Belelli et al., 2009; MacKenzie and Maguire, 2013).

Ganaxolone is an orally administered synthetic analogue of allopregnanolone and has been shown to supress seizure activity in numerous acute and chronic animal models (Carter et al., 1997; Kaminski et al., 2003; Reddy and Rogawski, 2009; 2010; Saporito et al., 2019). In humans, Ganaxolone has most robust evidence for the management of seizures associated with CDKL5 deficiency, a rare DEE associated with early-onset seizures and developmental impairment, and for which ganaxolone is approved in the US. Following encouraging results in a small open-label study, adjunctive ganaxolone was found to produce a 30.7% reduction in major seizures compared to 6.9% for placebo in a randomised placebo-controlled trial (Knight et al., 2022). Although ganaxolone has also been trialled in refractory adult focal epilepsy the observed benefits have been modest, and a phase 3 study did not show a significant reduction in seizure frequency compared to placebo (Meng et al., 2023). In a small dose-finding study for management of refractory status epilepticus, administration of intravenous ganaxolone as third-line therapy showed encouraging results with no subjects requiring intravenous anaesthesia within 24 h after treatment (Vaitkevicius et al., 2022). Although over 50% of cases comprised non-convulsive status epilepticus, a phase 3 randomised placebo-controlled trial is underway in light of these findings. Beyond ganaxolone, ETX155 is another candidate neurosteroid with efficacy for suppressing seizures in rodent models of epilepsy, and has recently commenced Phase 1 trials in photosensitive epilepsy (Perucca et al., 2023a).

### 4.7 Other subtype selective GABA_A_ receptor modulators and exploratory therapies

The prospect of maximizing therapeutic effects associated with GABA_A_ receptor modulation whilst minimizing drug dependence and sedation has led to the development of several subtype-selective compounds with promising pre-clinical characteristics, some of which are undergoing clinical development. ENX 101 is a α2, α3 and α5 subtype selective positive allosteric modulator that was well-tolerated by subjects in a Phase 1 study and is now undergoing Phase 2 testing for refractory focal epilepsy (Castellano et al., 2020; Jankovic et al., 2021; Perucca et al., 2023b). KRM-II-81 is an imidazodiazepine that is also highly selective for α2 and α3 subunit containing GABA_A_ receptors. KRM-II-81 has shown efficacy that is comparable or superior to diazepam across both acute chemical and electrical seizure models, and chronic pharmaco-resistant rodent models, but has not yet undergone trials in humans (Witkin et al., 2022).

Beyond improved receptor selectivity, gene therapy looms as an important component of epilepsy therapy in the future, and several approaches in development are designed to augment GABA_A_ receptor transmission. ETX101 is a recombinant adeno-associated viral (AAV) vector containing a GABA regulatory element designed to upregulate SCN1A (encoding the voltage-gated sodium channel NaV1.1) and restore interneuron function in patients with Dravet Syndrome.

Similar to ETX101, STK-001 is an antisense oligonucleotide designed to enhance the generation of wild-type SCN1A mRNA transcripts to restore inhibitory function in Dravet Syndrome (Bialer et al., 2020; Wengert et al., 2022). In mouse models, intraventricular administration of STK001 was associated with restoration of parvalbumin-positive interneuron firing, reduced seizures, and prolonged survival (Han et al., 2020). Following promising findings in Phase 1/2a studies in children with Dravet Syndrome, an open label extension is underway with interim results demonstrating reductions in seizure frequency and improvements in cognition and behaviour.

## 5 Conclusion

Modulation of the GABA_A_ receptor has been a mainstay of the antiseizure pharmacological armamentarium for over a century, and older drugs such as barbiturates and classical benzodiazepines still retain their place in clinical practice in both resource-scarce settings and for the management of status epilepticus. The coming years will see the introduction of novel agents with subunit specificity and gene therapies (Table 1) that act to restore GABA_A_ receptor transmission to correct inhibitory deficits. These developments build upon advances in our understanding of the complexities of the GABAergic system, and represent important steps toward precision epilepsy treatments that may improve efficacy whilst mitigating the adverse effects often associated with older drugs.

**TABLE 1:** Summary of established and emerging GABAA active medications used in epilepsy.

Drug	Action on GABA_A_ receptors	Route of administration	Pharmacokinetics	Clinical use/Trial	Notable adverse effects and drug interactions	References
Phenobarbital	Synaptic and extrasynaptic GABA_A_ receptor PAM via non-benzodiazepine binding site. GABA_A_ receptor agonist at higher doses	Intravenous	Half-life 50–150 h	Refractory status epilepticus	Somnolence, lethargy	Brodie and Kwan (2012), Nolan et al. (2013)
		Oral (primidone metabolised to phenobarbital)	Elimination via hepatic metabolism (predominantly CYP2C9) and renal excretion (up to 25%)	Focal and generalised epilepsy in resource poor settings	Reduced plasma levels of drugs metabolised by CYP450 system	
1,4 benzodiazepines (midazolam, diazepam, lorazepam, Clonazepam)	Synaptic GABA_A_ receptor PAM.	Intravenous, intramuscular (midazolam), oral, buccal (midazolam), intranasal (midazolam, lorazepam), rectal (diazepam)	Half-life 1.5–3 h (midazolam), 8–25 h (lorazepam), 24–48 h (diazepam). Diazepam converted to active metabolite N-Desmethyldiazepam. Eliminated via hepatic metabolism	Rescue medication in prolonged generalised and focal seizures, status epilepticus. Clonazepam: add-on drug for maintenance of focal and generalised epilepsies	Sedation, dizziness, ataxia, tolerance and drug dependence	Kienitz et al. (2022)
Clobazam (1,5 benzodiazepine)	Synaptic GABA_A_ receptor PAM via benzodiazepine binding site. Preferential selectivity for α2 subunit containing receptors	Oral	Converted to active metabolite N-desmethylclobazam via CYP450 system. Half-life 10–30 h (clobazam), 36–48 h (N-desmethylclobazam). Elimination via hepatic metabolism	Add-on therapy for refractory focal and generalised epilepsies. Lennox-Gastaut Syndrome	Sedation, dizziness, ataxia, tolerance and drug dependence (thought less compared to 1,4 benzodiazepines)	Conry et al. (2014)
Vigabatrin	Irreversible GABA-transaminase inhibitor	Oral	Half-life 5–7 h. Eliminated via renal excretion	Infantile epileptic spasms. Add-on therapy for refractory focal epilepsy	Peripheral visual field deficits (20%–25%)	Ben-Menachem (2011)
						Appleton and Montiel-Viesca (1993), Appleton et al. (1999)
Tiagabine	Inhibition of GABA re-uptake via preferential GAT1 blockade	Oral	Half-life 5–9 h. Eliminated via hepatic metabolism (predominantly CYP3A4)	Add-on therapy for refractory focal epilepsy	Dizziness, fatigue, tremor. Possible seizure exacerbation in idiopathic generalized epilepsies	Richens et al. (1995)
Valproate	Enhanced GABA synthesis via increased GAD activity. Reduced GABA breakdown via GABA transaminase blockade	Oral, Intravenous	Half-life 9–18 h. Eliminated via hepatic metabolism	First line therapy for idiopathic generalised epilepsies. Also effective for focal epilepsy. Status epilepticus	Tremor, sedation. High teratogenic risk. Increased plasma levels of antiseizure medications undergoing hepatic metabolism (e.g., lamotrigine, phenobarbital, carbamazepine)	Loscher, 1999 #235; Luder et al., 1990 #317}
Cenobomate	GABA_A_ receptor PAM, preferential activity at extrasynaptic receptors	Oral	Half-life 50–60 h	Refractory focal epilepsy	Somnolence, dizziness	Sharma et al. (2020)
Stiripentol	Synaptic and extrasynaptic GABA_A_ receptor PAM, preferential activity for α3 subunit containing receptors	Oral	Half-life 4.5–13 h. Eliminated via hepatic metabolism	Dravet Syndrome. Childhood focal epilepsies	Increased plasma levels of antiseizure medications metabolised via CYP450 system (e.g., clobazam, phenobarbital and phenytoin)	Nickels and Wirrell (2017)
Darigabat	GABA_A_ receptor PAM with preferential activity at α2, α3 and α5 subunit containing receptors	Oral	Half-life ∼11 h. Eliminated via hepatic metabolism	Undergoing Phase II testing for refractory generalised and focal seizures Efficacy in photosensitive epilsepy (small trial)	Somnolence, fatigue, headache	Engin et al. (2018), Owen et al. (2019), Cerne et al. (2022)
Padsenovil	GABA_A_ receptor partial agonist and PAM with preferential activity at α1 and α5 subunit containing receptors. SV2 modulation	Oral	Half-life 6–7 h. Eliminated via hepatic metabolism (CYP450 system)	No reduction in seizure frequency for refractory focal epilepsy in phase 3 trial	Somnolence, dizziness, headache	Rademacher et al. (2022)
Alprazolam (1,4 benzodiazepine)	Synaptic GABA_A_ receptor PAM.	Intranasal via Staccato device	Half-life 9–16 h. Eliminated via hepatic metabolism (CYP3A4)	Undergoing phase 3 testing as rescue medication in the outpatient setting	Cough, dysgeusia, oral dysesthesia, sedation somnolence	French et al. (2019), French et al. (2023)
Ganaxolone (neurosteroid)	Synaptic and extrasynaptic GABA_A_ receptor PAM, preferential activity at δ subunits containing receptors	Oral	Half-life 37–70 h. Eliminated predominantly via hepatic metabolism (CYP3A4)	Epilepsy associated with CDKL5 deficiency. Undergoing phase 3 testing in refractory status epilepticus	Somnolence, dizziness, fatigue	Knight et al. (2022)
ETX155 (neurosteroid)	Synaptic and extrasynaptic GABA_A_ receptor PAM	Oral	Half-life ∼24 h	Undergoing phase 1b testing in refractory focal epilepsy	Somnolence, fatigue, dizziness, headache	Perucca et al. (2023b)
ENX 101	GABA_A_ receptor PAM selective for α2, α3 and α5 subunit containing receptors	Oral	Half-life ∼20 h	Undergoing phase 2 testing in refractory focal epilepsy	Somnolence	Jankovic et al. (2021)
KRM-II-81	GABA_A_ receptor PAM selective for α2 and α3 subunit containing receptors	Oral (presumed)	*In vivo* data not available	Efficacy in pre-clinical animal models only	Data not available	Witkin et al. (2022)
STK-001	Antisense oligonucleotide designed to upregulate *SCN1A* and expression of NaV1.1 and restore function of GABAergic interneurons	Intrathecal injection	Data not available	Undergoing phase 2 testing in Dravet Syndrome MONARCH trial ADMIRAL trial	Vomiting, irritability, raised cerebrospinal fluid protein	Street et al. (2023)
ETX101	Upregulation of *SCN1A* and increased GABAergic interneuron function via AAV-vector based gene therapy	Intra-cerebroventricular injection	Human data not available	Phase 1 and 2 trials planned for Dravet Syndrome Inreased *SCN1A* mRNA transcripts in GABAergic internueorns in *Scn1a* ^*+/−*^ mice and primates	Human data not available	Tanenhaus et al. (2022)

Abbreviations: PAM, positive allosteric modulator; GAD, glutamic acid decarboxylase; IV, intravenous; SV2, synaptic vesical protein 2.
